# Probabilistic Fatigue/Creep Optimization of Turbine Bladed Disk with Fuzzy Multi-Extremum Response Surface Method

**DOI:** 10.3390/ma12203367

**Published:** 2019-10-15

**Authors:** Chun-Yi Zhang, Zhe-Shan Yuan, Ze Wang, Cheng-Wei Fei, Cheng Lu

**Affiliations:** 1 School of Mechanical and Power Engineering, Harbin University of Science and Technology, Key Laboratory of Advanced Manufacturing and Intelligent Technology, Ministry of Education, Harbin 150080, China; zhangchunyi@hrbust.edu.cn (C.-Y.Z.); yuanzheshan_ma17@hrbust.edu.cn (Z.-S.Y.); wangze_ma17@hrbust.edu.cn (Z.W.); 2 Department of Aeronautics and Astronautics, Fudan University, Shanghai 200433, China; 3 School of Aeronautics, Northwestern Polytechnical University, Xi’an 710072, China; lucheng2013@163.com

**Keywords:** fuzzy theory, multi-extremum response surface method, bladed disk, fatigue creep, probabilistic optimization

## Abstract

To effectively perform the probabilistic fatigue/creep coupling optimization of a turbine bladed disk, this paper develops the fuzzy multi-extremum response surface method (FMERSM) for the comprehensive probabilistic optimization of multi-failure/multi-component structures, which absorbs the ideas of the extremum response surface method, hierarchical strategy, and fuzzy theory. We studied the approaches of FMERSM modeling and fatigue/creep damage evaluation of turbine bladed disks, and gave the procedure for the fuzzy probabilistic fatigue/creep optimization of a multi-component structure with FMERSM. The probabilistic fatigue/creep coupling optimization of turbine bladed disks was implemented by regarding the rotor speed, temperature, and density as optimization parameters; the creep stress, creep strain, fatigue damage, and creep damage as optimization objectives; and the reliability and GH4133B fatigue/creep damages as constraint functions. The results show that gas temperature *T* and rotor speed *ω* are the key parameters that should be controlled in bladed disk optimization, and respectively reduce by 85 K and 113 rad/s after optimization, which is promising to extend bladed disk life and decrease failure damages. The simulation results show that this method has a higher modeling accuracy and computational efficiency than the Monte Carlo method (MCM). The efforts of this study provide a new useful method for overall probabilistic multi-failure optimization and enrich mechanical reliability theory.

## 1. Introduction

Mechanical structures are usually assembled by a several components; for example, the rotor system of an aero engine is assembled by a spindle, disk, blade, and other components [1]. If we directly establish the reliability optimization design model of an overall structure involving multi-material, multi-disciplinary, and multi-physics structures, the computational burden will become very large in analysis, so that computational efficiency is unacceptable [2]. Therefore, it is significant to propose an efficient method for an overall reliability optimization design of multi-component and multi-failure modes, to make computational precision and efficiency satisfy engineering requirements.

Recently, numerous methods on structural reliability optimization design have emerged [3,4,5]. The response surface method (RSM) is widely used in reliability optimal design for high efficiency and precision. Zhang et al. [6] firstly proposed an extremum response surface method to complete the reliability optimization of a two-link flexible manipulator; Fei et al. [7,8,9] studied an importance degree model with the extremum response surface method for the dynamic reliability optimization design of a mechanical assembly relationship such as turbine blade-tip radial clearance. However, the traditional RSM can’t meet the reliability optimization design of complex mechanical structures in modeling accuracy and simulation efficiency. To solve this issue, advanced response surface methods were developed recently. Song et al. [10] established a multiple response surface model by using back propagation-artificial neural network to construct a limit state function and establish a multi-objective reliability-based optimization model with a dynamic multi-objective particle swarm optimization algorithm for a reliability optimization design of an aero-engine blisk under fluid–thermal–structure coupling. Hamzaoui et al. [11] proposed an integrated method for estimating the resonance stress of blades with super high strength by combining the inverse of artificial neural network inverse (ANNI) with the Nelder–Mead optimization method. Rodríguez et al. [12] applied a probabilistic design procedure to a group of 10 blades of a low pressure (LP) stage steam turbine of 110 MW, in order to compute the stress changes and reliability due to variations in: damping, natural frequencies, vibration magnitude, and density. The computed vibration stresses were analyzed by applying probability distributions and statistical parameters of input and output to compute the useful life. Wang et al. [13] introduced evidence variables and fuzzy variables to describe cognitive uncertainty parameters and presented a novel dual-stage reliability analysis framework where the first stage incorporates the evidence information by the belief and plausibility measures and the second stage incorporates the fuzzy information by a membership function-like formula. Gao et al. [14] proposed an accurate and efficient fatigue prognosis based on a distributed collaborative response surface method, a substructure-based distributed collaborative probabilistic analysis method (SDCPAM), and a substructure analysis method. Ai et al. [15] discussed a probabilistic framework for fatigue reliability analysis. These works implement reliability-based optimization for many analytical objectives, through analyzing the submodels and then processing the response of submodels to carry out the overall design and analysis. The basic thought in the above works for handling multi-objective design problems provides an enlightened insight to reveal the overall reliability-based optimization design of turbine bladed disks with many failure modes, such as stress failure, strain failure, fatigue damage, creep damage, and so forth. However, since scientific research has its own development laws, the reliability optimization design was carried out in one failure mode at that time, without considering the correlation between the failure modes, and the fuzziness of the constraint boundary conditions.

Most works on aero-engine turbine blades regard the randomness of variables (parameters). Alongside the randomness, actually, some parameters in blade models, such as density, temperature, elastic modulus, boundary conditions, and so forth, possess obvious fuzziness centering on a certain value [16]. In fact, the probabilistic fatigue/creep optimization design of turbine bladed disks involves an obvious fuzziness for design parameters and constraint conditions as well as the coupling among many failure modes such as stress failure, strain failure, creep damage, fatigue damage, and so on [15,16,17,18]. Meanwhile, the fuzziness and coupling seriously negatively influence the design precision and efficiency of multi-object optimization when the above methods are directly applied. Therefore, it is urgent to propose an effective method for multi-object reliability-based optimization, in which the fuzziness for design parameters and constraint conditions as well as the coupling among many failure modes are fully considered in order to improve the modeling accuracy and simulation efficiency.

The objective of this paper is to attempt to propose a fuzzy multi-extremum response surface method (FMERSM) regarding failure correlation and parameter fuzziness, to improve the accuracy and efficiency of the overall dynamic reliability optimization design for a multi-component structure with multi-failure mode, by reasonably handling the transients. Then, the probabilistic fatigue/creep optimization design of an aero-engine bladed disk was effectively implemented with respect to this method, and the developed FMERSM is validated by a comparison of methods.

The remainder of this paper is organized as follows. The fuzzy multi-extremum response surface method (FMERSM) is studied in Section 2, comprising the FMERSM modeling approach, fatigue/creep theory, and the basic thought of the comprehensive probabilistic optimization of a bladed disk with FMERSM. Section 3 implements the fuzzy reliability-based optimization of bladed disk fatigue/creep damage including a parameters selection, finite element (FE) modeling, surrogate modeling, probabilistic fatigue/creep analysis, and method validation. In Section 4, some main conclusions are summarized.

## 2. Methods and Models

The extremum response surface method (ERSM) was firstly developed to simplify the modeling complexity for the transient probabilistic design of mechanical structures by considering the extreme values of the response process in sample extraction [6]. ERSM has been validated to have high-computational efficiency and acceptable accuracy relative to RSM, in the probabilistic design and optimization of aerospace structures/components [7,8,19,20,21,22]. The multi-extremum response surface method was proposed to handle the multi-model problem in the transient probabilistic analysis of multi-component structures, multi-discipline, and multi-failure modes by assimilating ERSM [19,23,24,25]. In most of the structural probabilistic designs, in fact, influential parameters and constraint conditions hold obvious fuzziness and seriously influence design precision. Therefore, it is reasonable to consider the fuzziness of design parameters and constraint conditions to improve the probabilistic design of structures, especially with multi-failure modes or multi-component structures. In respect of the heuristic thought of MERSM, this paper develops FMERSM with the consideration of fuzzy parameters and constraints to implement the fuzzy reliability-based optimization of bladed disk fatigue/creep damage.

### 2.1. FMERSM Modeling

Assuming that a structure system includes *m* components and one component has *n* failure modes (*m*,*n*∈**Z**) (the sample number of failure models is assumed in this study), as well as ***X****^ij^* indicating the input random variables of the *j*th failure mode in the *i*th component (for instance, the creep failure of a blade in a bladed disk system) and *y*^(*ij*)^(*t*,***X***^(*ij*)^) is the corresponding output response, enough of a data set {*y^ij^*_max_(*t*, ***X***^(*ij*)^): *j*∈***Z*_+_**} consisting of the maximum output responses of *y*^(*ij*)^(*t*,***X***^(*ij*)^) in the time domain is employed to fit the extremum output response *y* [24]:

(1) $$y=f\left(X\right)={\left\{y_{\max}^{\left(ij\right)}\left(X^{\left(ij\right)}\right)\right\}}_{i=1,2,\cdots ,m;j=1,2,\cdots ,n}$$

When the quadratic polynomials are considered, Equation (1) is rewritten as:

(2) $$y=a_{0}+BX+X^{T}CX$$

Regarding the fuzziness and randomness of data in Equation (2), the model comprising numerous sub-models (${\tilde{y}}_{\max}^{\left(11\right)},\;{\tilde{y}}_{\max}^{\left(12\right)},\;\cdots ,\;{\tilde{y}}_{\max}^{\left(i1\right)},{\tilde{y}}_{\max}^{\left(i2\right)},\cdots ,{\tilde{y}}_{\max}^{\left(ij\right)}$), the FMERSM model, for multi-failure structure, can be structured as:

(3) $$\left\{ \begin{array}{l} {\tilde{y}}_{\max}^{\left(11\right)}=f\left(X^{\left(11\right)}\right)={\tilde{A}}_{0}^{\left(11\right)}+{\tilde{B}}^{\left(11\right)}X^{\left(11\right)}+{\left(X^{\left(11\right)}\right)}^{T}{\tilde{C}}^{\left(11\right)}X^{\left(11\right)}\\ {\tilde{y}}_{\max}^{\left(12\right)}=f\left(X^{\left(12\right)}\right)={\tilde{A}}_{0}^{\left(12\right)}+{\tilde{B}}^{\left(12\right)}X^{\left(12\right)}+{\left(X^{\left(12\right)}\right)}^{T}{\tilde{C}}^{\left(12\right)}X^{\left(12\right)}\\ \vdots \\ {\tilde{y}}_{\max}^{\left(ij\right)}=f\left(X^{\left(ij\right)}\right)={\tilde{A}}_{0}^{\left(ij\right)}+{\tilde{B}}^{\left(ij\right)}X^{\left(ij\right)}+{\left(X^{\left(ij\right)}\right)}^{T}{\tilde{C}}^{\left(ij\right)}X^{\left(ij\right)} \end{array}\right.$$

in which ${\tilde{X}}^{\left(ij\right)}$ is the fuzzy random input variable vector of the *j*th failure mode in the *i*th component, and ${\tilde{y}}_{\max}^{\left(ij\right)}$ is the corresponding extremum output response. ${\tilde{A}}_{0}^{\left(ij\right)},{\tilde{B}}^{\left(ij\right)}$ and ${\tilde{C}}^{\left(ij\right)}$ are the constant term, linear term, and quadratic term of the *j*th failure mode in the *i*th component, respectively. ${\tilde{B}}^{\left(ij\right)},{\tilde{C}}^{\left(ij\right)}$ and ${\tilde{X}}^{\left(ij\right)}$ are denoted by:

(4) $${\tilde{B}}^{\left(ij\right)}=\left[b_{1}^{ij},b_{2}^{ij},\cdots ,b_{k}^{ij}\right]$$

(5) $${\tilde{C}}^{\left(ij\right)}=\left(\begin{array}{ccc} c_{11}^{\left(ij\right)} & \ldots & 0\\ \vdots & \ddots & \vdots \\ c_{k1}^{\left(ij\right)} & \cdots & c_{kk}^{\left(ij\right)} \end{array}\right)$$

(6) $${\tilde{X}}^{\left(ij\right)}={\left[X_{1}^{\left(ij\right)},X_{2}^{\left(ij\right)},\cdots ,X_{k}^{\left(ij\right)}\right]}^{T}$$

where $b_{m}^{ij},c_{mn}^{\left(ij\right)},X_{m}^{\left(ij\right)}$(*m*, *n* = 1, 2, …, *k*) are elements (or components) in ${\tilde{B}}^{\left(ij\right)}$,${\tilde{C}}^{\left(ij\right)}$ and ${\tilde{X}}^{\left(ij\right)}$ respectively.

The modeling process of Equation (3) regards the randomness and fuzziness of design parameters and constraints based on FMERSM. Therefore, this model (Equation (3)) is called a FMERSM model in this paper.

### 2.2. Fatigue/Creep Modeling for Probabilistic Optimization of Bladed Disks

Under fatigue/creep coupling failure mode, this paper adopts FMERSM to complete the fuzzy probabilistic fatigue/creep optimization of bladed disks. For a structure system with *m* components, ${\tilde{x}}_{i}$ indicates the fuzzy optimization parameters of the *i*th component. The main plan is to minimize the objective function $f\left({\tilde{x}}_{1},{\tilde{x}}_{2},\cdots ,{\tilde{x}}_{n}\right)$ subject to the overall reliability performance *R*(*x*, *w, D*_c_, *D_f_*) and coupling critical damage *D_cr_*, which is a single-objective constrained optimization problem. The sub-plan is to maximize the reliability *R_i_* ($R_{i}=R\left(R_{i}^{\left(1\right)},R_{i}^{\left(2\right)},\cdots ,R_{i}^{\left(k\right)}\right)$) of the *i*th component subject to mechanical load and constraints, which is a multi-objective constrained optimization problem. By introducing pseudo-variables [26], the cyclic optimization between the main plan and sub-plans is done until the convergence condition is satisfied. The fuzzy probabilistic optimization model is shown in Equation (7).

(7) $$\left\{\begin{array}{cc} \; & \mathrm{find}\;\tilde{x}={\left(x_{1},x_{2},\cdots ,x_{n}\right)}^{T}\\ \; & \min\;f\left({\tilde{x}}_{1},{\tilde{x}}_{2},\cdots {\tilde{x}}_{n}\right)=E\left\{\sum_{i=1}^{l}f_{i}\left({\tilde{x}}_{i}\right)\right\}\;\overset{{\tilde{x}}_{i}}{\to }\;\\ \mathrm{subject}\;\mathrm{to} & \left\{\begin{array}{c} R\left(x,\omega ,D_{c},D_{f}\right)=R\left(R_{1},R_{2}\cdots R_{m}\right)\geq R_{0}\;\overset{R_{i}}{\leftarrow }\;\\ D_{c}+D_{f}\leq D_{cr} \end{array}\right. \end{array}\right.\left\{\begin{array}{cc} \; & \mathrm{find}\;{\tilde{x}}_{i}=\left[x_{i1},x_{i2},\cdots ,x_{in}\right]\\ \; & \max\;R_{i}=R\left(R_{i}^{\left(1\right)},R_{i}^{\left(2\right)}\cdots R_{i}^{\left(k\right)}\right)\\ \mathrm{subject}\;\mathrm{to} & \left\{\begin{array}{c} {\tilde{g}}_{j}\left(x_{j}\right)\subseteq {\tilde{G}}_{j}\\ x_{i1}^{L}\leq x_{i}\leq x_{in}^{U} \end{array}\right. \end{array}\right.$$

where ${\tilde{x}}_{i}$ is the *i*th design variable; and *w* is the random parameters of mechanical load and material property. $x_{i1}^{L},x_{in}^{U}$ represent the lower and upper limit of the *i*th fuzzy design variables; *D_c_* is the total amount of creep damages; *D_f_* is the total amount of fatigue damages; *D_cr_* is fatigue–creep coupled critical damage; ${\tilde{g}}_{j}\left(x_{i}\right)$ denotes the stress and deformation of a component; and ${\tilde{G}}_{j}$ is the allowable range of ${\tilde{g}}_{j}(x_{i})$. By the *λ* level-cut method, the fuzzy subset ${\tilde{G}}_{j}$ is decomposed into the common set *G_j_*(λ^*^), as explained in Equation (8); then, the problem of fuzzy probabilistic constrained optimization can be transformed into the conventional probabilistic optimization design problem [27].

(8) $$G_{j}\left(\lambda^{*}\right)=\left\{g|u_{{\tilde{G}}_{j}}\left(g\right)\geq \lambda^{*},j=1,2,\cdots ,J\right\}$$

where $u_{{\tilde{G}}_{j}}(g)$ is allowable constraint of the *j*th component stress and deformation; and λ* is optimal horizontal cut set.

### 2.3. Miner Linear Accumulation Damage Law

Under the interaction between fatigue and creep, the overall damage of the structure is equal to the sum of fatigue damage and creep damage, which is the Miner linear cumulative damage law [28] as follows:

(9) $$\left\{ \begin{array}{l} D_{f}+D_{c}\leq D_{cr}\\ D_{f}=\sum_{j=1}^{nf}\frac{n_{j}}{N_{jf}}\\ D_{c}=\sum_{i=1}^{nc}\frac{t_{i}}{T_{ic}} \end{array}\right.$$

in which *n_f_* is the number of stresses acting on a component; *n_j_* is the number of cycles acted by the *j*th stress; *N_jf_* is the fatigue life under the *j*th acting stress; *n_c_* is the number of stress levels; *t_i_* is the hold time of the *i*th stress; and *T_ic_* is the creep failure time of the *i*th stress.

When the structure is destroyed (*D_cr_* = 1), the relationship between *D_f_* and *D_c_* [29] is:

(10) $$D_{f}=F\left(D_{c}\right)=2-e^{\theta_{1}D_{c}}+\frac{e^{\theta_{1}}-2}{e^{-\theta_{2}}-1}\left(e^{-\theta_{2}D_{c}}-1\right)$$

where *θ*_1_ and *θ*_2_ are fatigue–creep characteristic parameters.

The strain fatigue life prediction model is used to predict the low-cycle fatigue life.

(11) $$\frac{\Delta \varepsilon }{2}=\frac{\sigma_{f}}{E}{\left(2N_{f}\right)}^{b}+\varepsilon_{f}{\left(2N_{f}\right)}^{c}$$

in which Δε is the amplitude of total strain; $N_{f}$ is the fatigue life; $\sigma_{f}$ is the fatigue strength coefficient; $\varepsilon_{f}$ is the fatigue ductility coefficient; b is the fatigue strength index; and c is the fatigue ductility index.

The creep life prediction equations commonly used in material manuals include creep life prediction equations and thermal strength parameter synthesis equations. The persistence equation is expressed in the form of the thermal intensity parameter synthesis equation.

(12) $$\mathrm{lg}\sigma =a_{0}+a_{1}p+a_{2}p^{2}+a_{3}p^{3}$$

(13) $$p=\left(\mathrm{lg}t_{i}+c\right)C\;(i=0,\;1,\;2,\;3)$$

in which σ is durable strength; $a_{r}\left(r=0,\;1,\;2,\;3\right)$ is the undetermined coefficient in which *r* indicates the subscript of the *r*th coefficient in Equation (12); p is the thermal intensity parameter; *t_i_* is the hold time of the *i*th stress; and *c* and *C* are the constants related to fatigue ductility and temperature, respectively, which were generally gained by experiments.

### 2.4. Basic Thought of Probabilistic Fatigue/Creep Optimization with FMERSM

**Figure 1:**
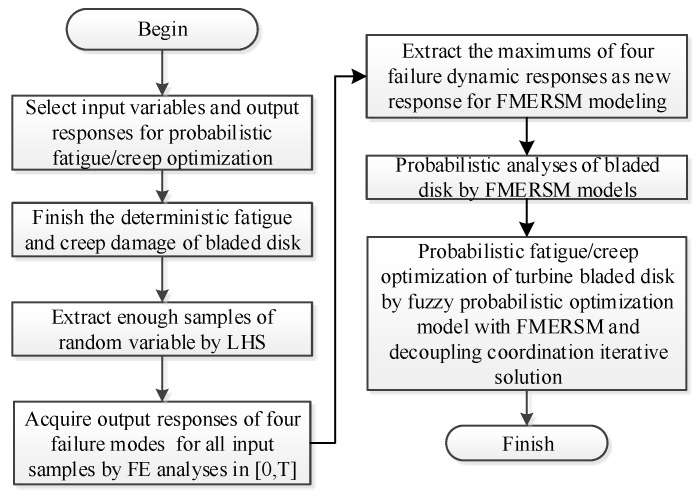
Flow chart of reliability optimization based on the fuzzy multi-extremum response surface method (FMERSM) method.

The basic thought of probabilistic fatigue/creep optimization with FMERSM is illustrated below. (1) Regard material density, gas temperature, pneumatic pressure, elastic modulus, and thermal expansion coefficient as input variables, and the maximum creep stress, maximum creep strain, maximum fatigue damage, and maximum creep damage as output responses. (2) Carry out the deterministic analysis of a bladed disk based on FE models with the consideration of design parameters. (3) Obtain the fatigue damage and creep damage of a bladed disk under each load by the fatigue–creep damage equation of GH4133B discussed in Section 2.2 and Miner linear damage accumulation law introduced in Section 2.3. (4) Considering the randomness and fuzziness of input variables, enough samples of input random variables are extracted by the Latin hypercube sampling technique [30]. (5) Calculate the dynamic responses of bladed disk creep stress, creep strain, creep damage, and fatigue damage in the time domain for all input samples by FE models, and extract the maximum values of dynamic output responses as new output responses to establish the FMERSM function. (6) Perform a probabilistic analysis of a bladed disk based on the FMERSM function. (7) Complete the probabilistic fatigue/creep optimization of a turbine bladed disk by the fuzzy probabilistic optimization model with FMERSM and decoupling coordination iterative solution. The flowchart is shown in Figure 1.

## 3. Fuzzy Probabilistic Fatigue/Creep Optimization of Turbine Bladed Disk

In this section, the fuzzy probabilistic fatigue/creep optimization of a turbine bladed disk is performed with respect to the proposed FMERSM and established probabilistic optimization model in Section 2.

### 3.1. Parameters Preparation

**Figure 2:**
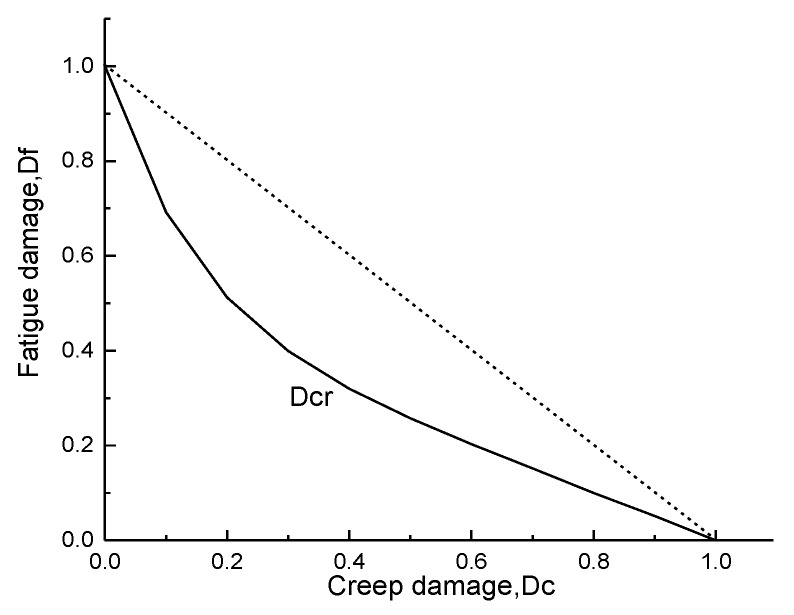
Curves of GH4133B fatigue–creep damage.

With respect to the material test, the fatigue/creep material parameters *θ*_1_ and *θ*_2_ of an aero-engine turbine bladed disk with a GH4133B superalloy at the temperature of 600 °C and experimental load of 18 KN is 0.36 and 6.5, respectively. The fatigue–creep damage curve of GH4133B superalloy (Ni–Cr-based precipitation hardening-type deformation high-temperature alloy) is shown in Figure 2. In this study, we selected a 1/40 turbine bladed disk of an aero-engine as the object of study, and a GH4133B superalloy as the material of the bladed disk [31]. Density *ρ*, rotational speed *ω*, temperature *T*, pneumatic pressure *p*, elastic modulus *E*, and thermal expansion coefficient *α* are considered as fuzzy variables. Moreover, in respect of engineering practice, the length of the fuzzy region is defined as 0.05 times the mean value, as shown in Table 1. The parameters in Table 1 are assumed to obey normal distribution, and are mutually independent.

### 3.2. Deterministic Analysis of Bladed Disk

**Figure 3:**
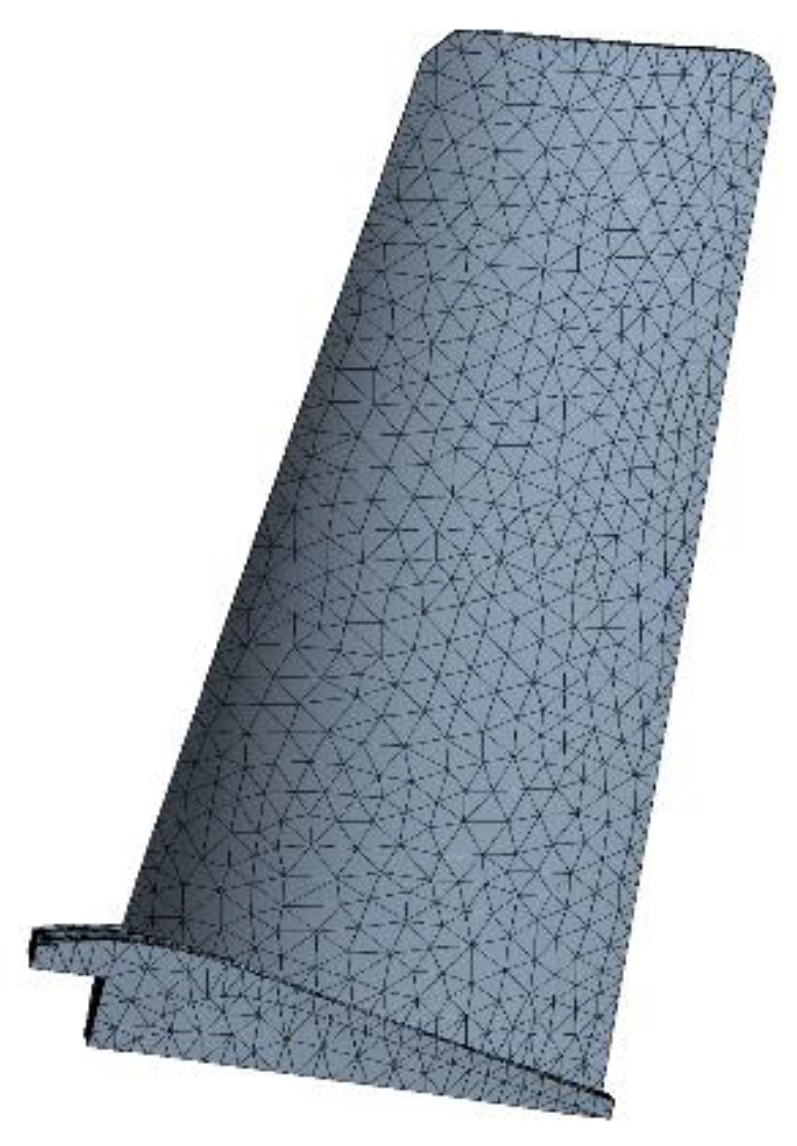
Finite element model of the blade.

**Figure 4:**
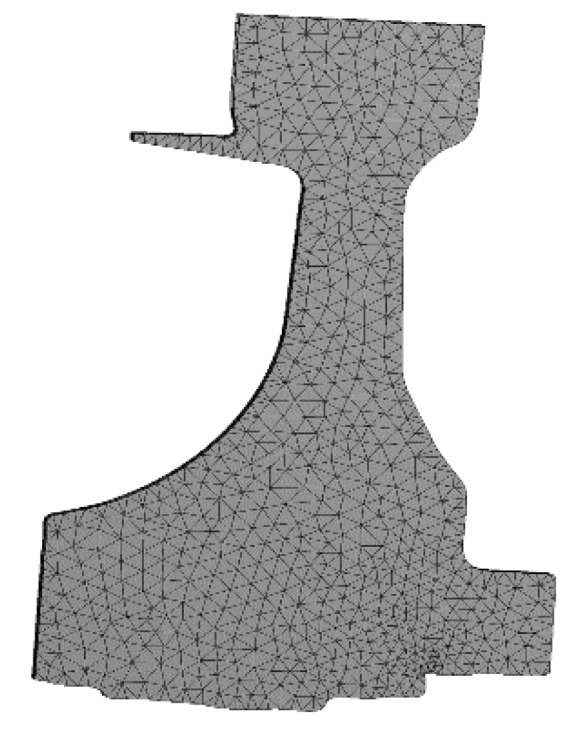
Finite element model of the disk.

**Figure 5:**
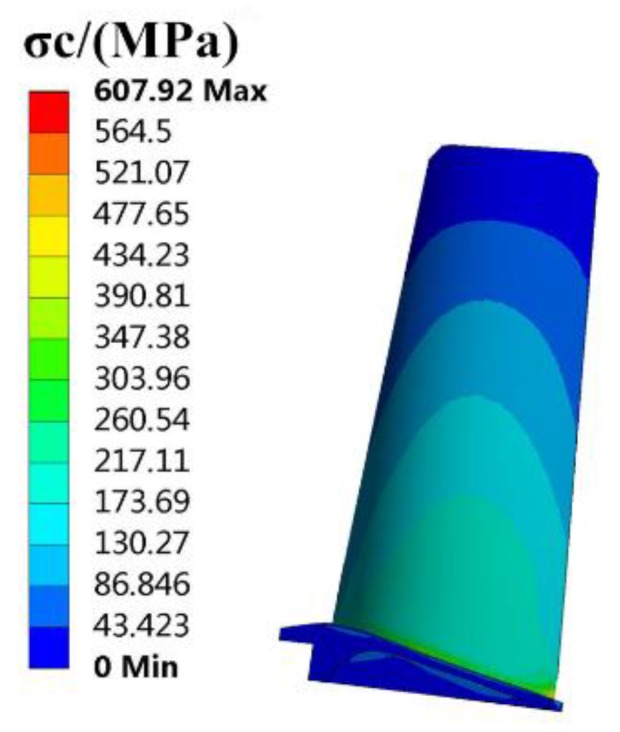
Distribution of blade creep stress.

**Figure 6:**
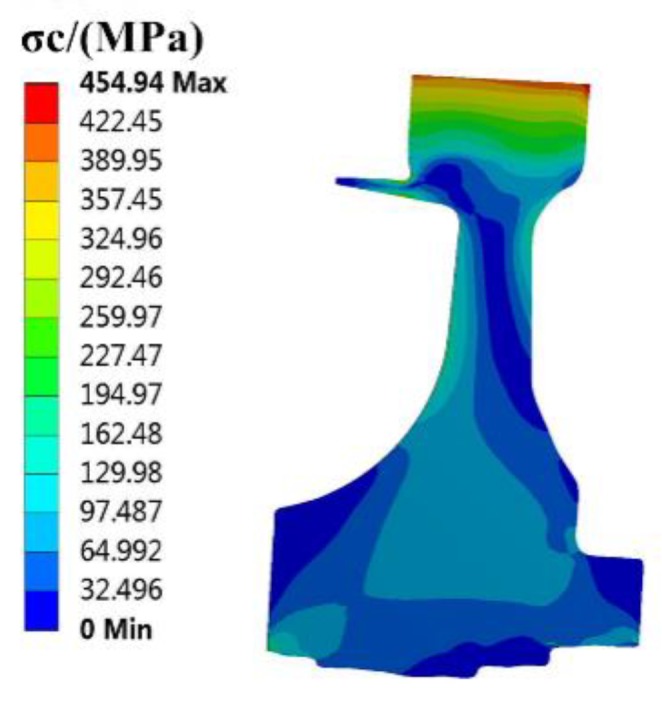
Distribution of disk creep stress.

**Figure 7:**
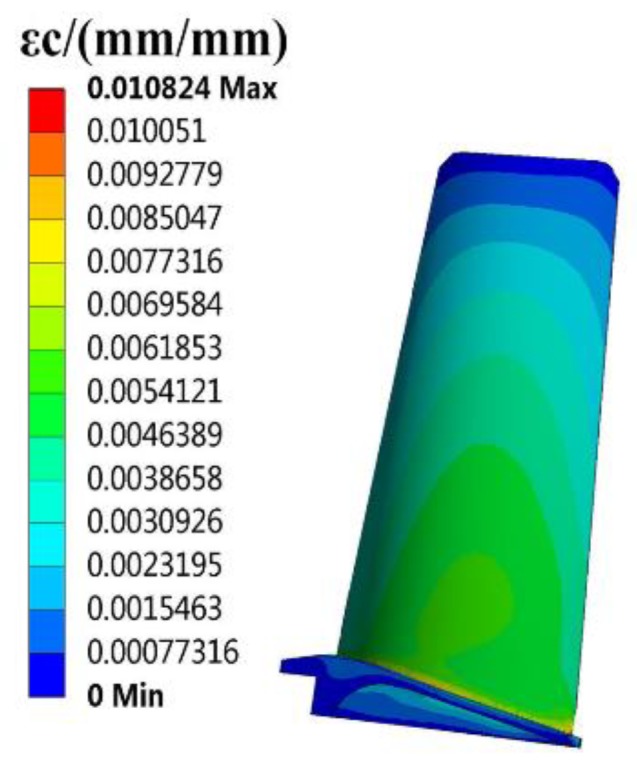
Distribution of blade creep strain.

**Figure 8:**
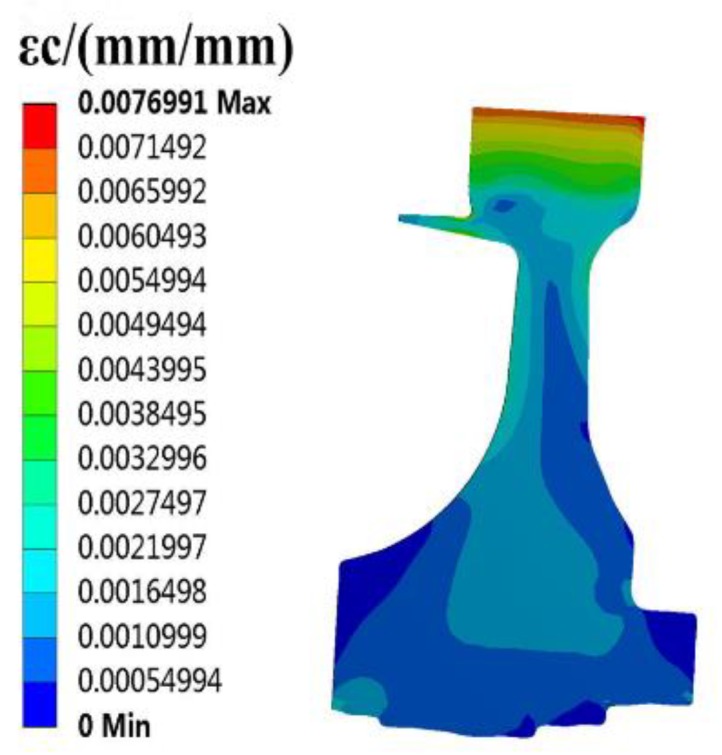
Distribution of disk creep strain.

The finite element (FE) models of the blade and disk are shown in Figure 3 and Figure 4. The FE model of the blade consists of tetrahedrons with 39,547 elements, and the FE model of the disk consists of tetrahedrons with 58,271 elements. The number of cells (meshes) is required by the convergence analyses of the responses [15,32,33]. The FE basic equations of the bladed disk comprising a shape function of the tetrahedron in Equation (14) [34], geometric equation in Equation (15) [35], physical equation in Equation (16) [36], and Norton implicit creep equation in Equation (17) [37] are analyzed with regard to the means of the parameters in Table 1. From this analysis, the distributions of the creep stress and creep strain of the bladed disk are drawn in Figure 5, Figure 6, Figure 7 and Figure 8. As seen in Figure 5, Figure 6, Figure 7 and Figure 8, the maximum creep stress and maximum creep strain of the bladed disk are at the blade-root and disk tenon groove tip, respectively.

(14) $$N_{i}=\frac{1}{6v}\left(a_{i}+b_{i}x+c_{i}y+d_{i}z\right),\;i=1,2,3,4$$

(15) $$\left\{ \begin{array}{l} \varepsilon_{x}=\frac{\partial u}{\partial x},\varepsilon_{y}=\frac{\partial v}{\partial y},\varepsilon_{z}=\frac{\partial w}{\partial z}\\ \gamma_{xy}=\frac{\partial u}{\partial y}+\frac{\partial v}{\partial x},\gamma_{yz}=\frac{\partial v}{\partial z}+\frac{\partial w}{\partial y},\gamma_{zx}=\frac{\partial w}{\partial x}+\frac{\partial u}{\partial z} \end{array}\right.$$

(16) {σ}=[D]{ε}

(17) $$\varepsilon_{c}=c_{1}\sigma^{C_{2}}e^{-C_{3}/T}$$

where *v* is the volume of the tetrahedron; *a_i_*, *b_i_*, *c_i_* and *d_i_* are the related coefficients of node geometry; *ε_x_, ε_y_*, *ε_z_* and *γ_xy_*, *γ_yz_*, *γ_zx_* are the elastic line strains and shear strains along the *x*, *y* and *z* directions, respectively; [***σ***] = [*σ_x_*, *σ_y_*, *σ_z_*, *τ_xy_*, *τ_yz_*, *τ_zx_*] (*σ*-structural stress) and [*ε*] = [*ε_x_*, *ε_y_*, *ε_z_*, *γ_xy_*, *γ_yz_*, *γ_zx_*] are the stress and strain of the components; [***D***] is the elastic matrix; *c*_1_, *c*_2_, and *c*_3_ stand for experimental coefficients.

Based on the low-cycle load spectrum described in [34], the Miner linear cumulative damage law in Equation (9) and fatigue-creep damage relation in Equation (10) of GH4133B [38] were resolved by programming in MATLAB (R2017a) simulation environment. When the number of cyclic loads is 5530, the fatigue damage *D_f_* and creep damage *D_c_* of bladed disk under a cyclic load are shown in Table 2.

### 3.3. FMERSM Modeling

By the Latin hypercube sampling technique [30], 100 samples on fuzzy input random variables were extracted in respect of the max creep stress, max creep strain, max fatigue damage, and max creep damage of the bladed disk, to acquire model parameters and establish the FMERSM model in Equations (18) and (19).

(18) $$\left\{\begin{array}{cc} {\tilde{y}}^{\left(11\right)} & =-257.3754-0.0444\rho -0.1486\omega +2.3354\times 10^{3}T+1.1507p+1.4334\times 10^{-4}E-0.2077a\\ & +6.5536\times 10^{-4}\rho^{2}+0.0132\omega^{2}+3.6612\times 10^{5}T^{2}+0.0102p^{2}4.2577\times 10^{-5}E^{2}-4.8668\times 10^{-4}a^{2}\\ {\tilde{y}}^{\left(12\right)} & =0.0099-1.6579\times 10^{-6}\rho -1.3072\times 10^{-5}\omega -0.0599T-9.8795\times 10^{-6}p+1.4733\times 10^{-9}E-1.8500\times 10^{-6}a\\ & +2.8940\times 10^{-6}\rho^{2}+14.4810\omega^{2}+7.4616\times 10^{-7}T^{2}+5.3444\times 10^{-4}p^{2}+4.4308\times 10^{-6}E^{2}+3.7239\times {10^{-7}}^{2}a^{2}\\ {\tilde{y}}^{\left(13\right)} & =1.5805\times 10^{-4}-1.3288\times 10^{-8}\rho -1.0181\times 10^{-7}\omega -2.7108\times 10^{-4}T-6.7492\times 10^{-8}p+4.3108\times 10^{-12}E-1.3172\times 10^{-8}a\\ & +2.8445\times 10^{4}\rho^{2}+0.0027\omega^{2}+0.1975T^{2}-6.5354p^{2}-2.6096E^{2}+0.0043a^{2}\\ {\tilde{y}}^{\left(14\right)} & =3.0155\times 10^{-4}+3.2551\times 10^{-8}\rho -1.0601\times 10^{-7}\omega +0.0016T-7.4068\times 10^{-8}p+5.8844\times 10^{-12}E-1.2685\times 10^{-7}a\\ & +2.9205\times 10^{-5}\rho^{2}+6.7736\times 10^{-6}\omega^{2}+1.2254\times 10^{-4}T^{2}+1.8214\times 10^{-4}p^{2}-8.1228\times 10^{-7}E^{2}+6.6609\times 10^{-8}a^{2} \end{array}\right.$$

(19) $$\left\{\begin{array}{cc} {\tilde{y}}^{\left(21\right)} & =4.6236\times 10^{3}-0.0477\rho -0.3303\omega -2.4545\times 10^{3}T-9.4539p+1.9172\times 10^{-4}E-0.2481\alpha \\ & -23.2706\rho^{2}+0.0031\omega^{2}+11.0768T^{2}+5.9537\times 10^{-5}p^{2}+6.8958\times 10^{-4}E^{2}+3.8867\times 10^{-4}\alpha^{2}\\ {\tilde{y}}^{\left(22\right)} & =0.1404+1.4686\times 10^{-7}\rho -2.4194\times 10^{-5}\omega +0.1010T-3.1221\times 10^{-4}p+2.1079\times 10^{-9}E-1.5086\times 10^{-5}\alpha \\ & +2.7152\times 10^{-11}\rho^{2}-1.1026\times 10^{-11}\omega^{2}+5.5067\times 10^{-9}T^{2}+2.0951\times 10^{-9}p^{2}-8.5852\times 10^{-12}E^{2}-4.0029\times 10^{-11}\alpha^{2}\\ {\tilde{y}}^{\left(23\right)} & =-8.7299\times 10^{-5}-2.6642\times 10^{-9}\rho -5.0414\times 10^{-8}\omega -1.9935\times 10^{-4}T+3.5076\times 10^{-7}p+7.8942\times 10^{-12}E-3.7701\times 10^{-8}\alpha \\ & +4.0029\times 10^{-4}\rho^{2}+3.1495\times 10^{-6}\omega^{2}+7.2796\times 10^{-5}T^{2}+6.8958\times 10^{-4}p^{2}-9.4331\times 10^{-5}E^{2}+3.8867\times 10^{-4}\alpha^{2}\\ {\tilde{y}}^{\left(24\right)} & =0.0059+6.2600\times 10^{-8}\rho -1.0922\times 10^{-6}\omega +0.0140T-4.3691\times 10^{-6}p+3.2447\times 10^{-11}E+1.8682\times 10^{-6}\alpha \\ & +1.9739\times 10^{-9}\rho^{2}+3.6047\times 10^{-12}\omega^{2}+3.3291\times 10^{-10}T^{2}+0.0046p^{2}+0.0048E^{2}-7.3346\times 10^{-4}\alpha^{2} \end{array}\right.$$

The FMERSM models in Equations (18) and (19) are used to perform the probabilistic fatigue/creep optimization of a bladed disk involving sensitivity analysis and reliability analysis in the following subsection.

### 3.4. Probabilistic Fatigue/Creep Optimization of Bladed Disk

**Figure 9:**
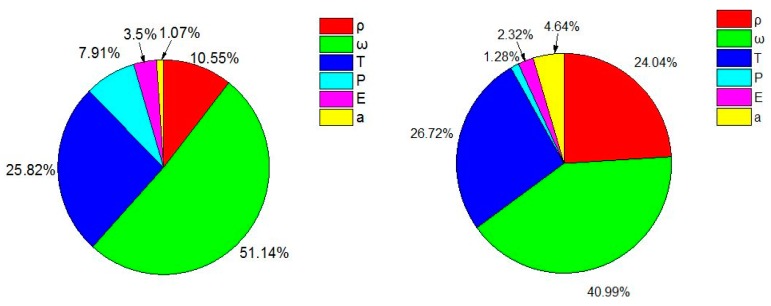
Sensitivity indexes of parameters on bladed disk coupling failure.

Regarding the FMERSM models in Equations (18) and (19), the reliability sensitivity index of input random variables for a bladed disk was obtained in Table 3 and Figure 9 by sensitivity analysis with MC simulation.

**Figure 10:**
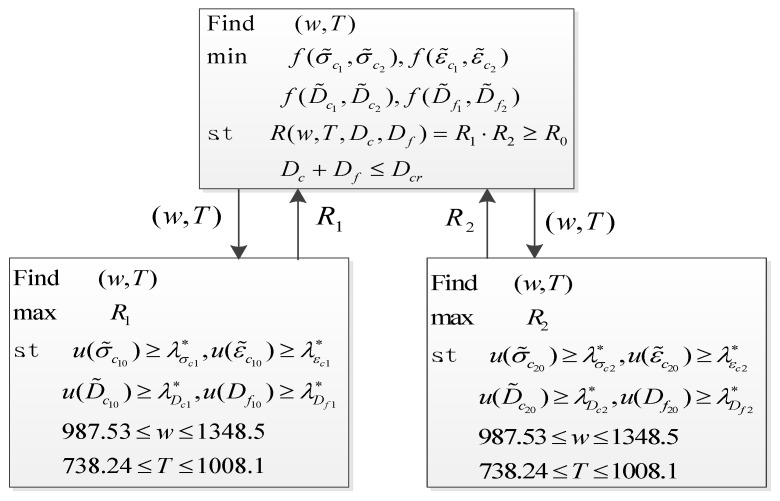
Fuzzy probabilistic fatigue/creep optimization model of a bladed disk.

As shown in the sensitivity analysis of a bladed disk, rotor speed *w* and temperature *T* are the main factors and greatly influence the coupling failure of a bladed disk as the two largest sensitivity degrees and effect probabilities, while the other parameters have little impact on the coupling failure of a bladed disk as smaller sensitivity degrees and effect probabilities. We extract high-sensitivity input random variables as design variables to conduct the fuzzy probabilistic fatigue/creep optimization of a bladed disk. In the main planning model, the reliability product of *R*_1_·*R*_2_ and the coupling critical damage *D_cr_* for the bladed disk are taken as the constraints. In the sub-planning model, the parameters (*ω*,*T*) with a high-sensitivity index are regarded as the design variables. The creep stress *σ_c_*, creep strain *ε_c_*, fatigue damage *D_f_*, creep damage *D_c_*, maximum blade reliability *R*_1_, and maximum disk reliability *R*_2_ were evaluated. The allowable comprehensive reliability of the bladed disk is *R*_0_ = 0.99. The optimal level λ* is solved by the fuzzy comprehensive evaluation method [13,23,39], and the substitution of λ* into the asymmetric fuzzy optimized conversion condition (Equation (8)). The allowable means of a bladed disk with the corresponding failure modes are shown in Table 4. The fuzzy probabilistic fatigue/creep optimization model of a bladed disk was established as illustrated in Figure 10, and the optimization models were solved by the MATLAB program and iteratively solved for all levels. The optimization results are listed in Table 5.

### 3.5. FMERSM Validation

To verify the effectiveness of FMERSM, the reliability-based optimization of a bladed disk was completed with MCM and FMERSM, based on the same variables in Table 1 and computing conditions. For dynamic probabilistic analyses under different simulations (10^2^, 10^3^, 10^4^, and 10^5^), the computing time and reliability degrees of a bladed disk are listed in Table 6. The optimization results of object functions under different simulations are listed in Table 7.

As shown in Table 6, the following conclusions were obtained from the probabilistic failure analysis of a bladed disk. (1) The MC method does not have computing time at 10^5^ simulations, because the MC method cannot perform the calculation for a too-large computational burden for a probabilistic analysis of bladed disk FE models. Thus, it is inefficient for the MC method to conduct the design analysis of a complex structure with large-scale simulations. (2) The time-cost for the probabilistic analysis of a bladed disk increases with the increase of MC simulations. (3) The time consumption of the FMERSM is far less than that of the MC method for the same number of simulations. For instance, the FMERSM only spends 0.437 s for 10,000 simulations, which is only about 1/10^6^ that of the MC method. Meanwhile, the strength of the FMERSM in time computation is more obvious with increasing simulations. Thus, it is demonstrated that the efficiency of the FMERSM is far higher than that of the MC method in calculation, and the FMERSM is an efficient approach replacing FE models for the probabilistic analysis of a complex structure with many components or multi-failure modes. (4) For the same simulations, the reliability degrees of bladed disk coupling dynamic failure probability with FMERSM are almost consistent with those of the MC method. Moreover, the reliability degree of the bladed disk increases and becomes higher with the rise of simulations. It is illustrated that more precise results such as the reliability degree can be gained by increasing the number of MC simulations against the response surface models, for structure design analysis from a probabilistic perspective.

As revealed in Table 7, summarized from the probabilistic fatigue/creep optimization of a bladed disk, the creep stress, creep strain, fatigue damage, and creep damage of the blade in respect of FMERSM are reduced by 19.9%, 18.93%, 31.64%, and 14.77%, respectively. Meanwhile, the MC method reduces the creep stress, creep strain, fatigue damage, and creep damage of the disk by 9.8%, 7%, 88%, and 0.47%, respectively. The comprehensive reliability index of the bladed disk was increased from 99.515% to 99.635%. It is verified that the FMERSM is workable for the fuzzy probabilistic fatigue/creep optimization of complex structures, similar to a turbine bladed disk.

In summary, the developed FMERSM has high modeling precision and simulation efficiency for the comprehensive reliability optimization design for multi-component structures with multi-failure modes.

## 4. Conclusions

The objective of this study is to develop a high-efficient reliability-based optimization method, called the fuzzy multi-extremum response surface method (FMERSM), for the probabilistic fatigue/creep coupling optimization of a turbine bladed disk. This paper has investigated the theory and modeling of FMERSM, and gives the procedure of probabilistic optimization of a multi-component structure with multi-failure modes for the fuzzy probabilistic fatigue/creep optimization of a turbine bladed disk with the considerations of the correlation of the failure modes and the fuzziness of the constraint boundary conditions. Through the works in this study, some conclusions are summarized as follows:

(1) With regard to the probabilistic failure analysis of a bladed disk, we find that the FMERSM costs less analytical time (0.437 s for 10,000 simulations), and thus has high computational efficiency relative to the Monte Carlo (MC) method (432,000 s for 10,000 simulations), but has an acceptable computational precision (99.77%) of the reliability degree, which is almost consistent with the FE method based on MC simulation with a reliability degree of 0.9983. Moreover, the strengths of the proposed FMERSM in modeling and simulation become more obvious with the increase of simulations. 

(2) In terms of the probabilistic fatigue/creep optimization of a bladed disk, it is illustrated that the developed FMERSM is more workable than the MC method. The reason is that the optimal parameters, including design parameters and optimization objects, are preferable by larger reductions (19.9%, 18.93%, 31.64%, and 14.77% for the creep stress, creep strain, fatigue damage, and creep damage of the blade, respectively), and a higher reliability degree of 99.635%.

The efforts of this paper provide a useful way for high-precise modeling and high-efficient simulation for the fuzzy comprehensive probabilistic optimization of multi-failure/multi-component structures, because the accuracy of the model is close to that of the MC method, while the calculation time is only 1/10^6^. Meanwhile, this work enriches the theory of mechanical reliability.

## Figures and Tables

**Table 1:** Distribution characteristics of input random variables.

Random Variables	Mean	Length of Fuzzy Zone	Distribution
Density, *ρ*, kg·m^−3^	8210	410.5	Normal
Rotor speed, *ω*, rad·s^−1^	1168	58.4	Normal
Temperature *T*, K	873.15	43.658	Normal
Pneumatic pressure *p*, MPa	0.1	0.005	Normal
Elastic modulus *E*, MPa	163,000	8150	Normal
Thermal expansion coefficient *α*, ×10^−6^ °C^−1^	9.4	0.47	Normal

**Table 2:** Results of bladed disk fatigue-creep damage.

	Fatigue Damage *D_f_*	Creep Damage *D_c_*
Blade	0.36363	0.0039
Disk	0.40859	0.0041

**Table 3:** Sensitivity index of a bladed disk.

Blade			Disk		
Variables	Sensitivity	Effect Probability %	Variable	Sensitivity	Effect Probability %
*ρ*	0.09855	10.55	*ρ*	0.22067	24.04
*ω*	0.477722	51.14	*ω*	0.386637	40.99
*T*	0.241222	25.82	*T*	0.252026	26.72
*p*	0.073895	7.91	*p*	–0.0121	1.28
*E*	0.032676	3		0.02191	2.32
*α*	−0.01	1.07	*α*	0.043787	4.64

**Table 4:** Optimal level threshold and allowable mean of a bladed disk.

Blade				Disk			
Optimal Level Threshold		Allowable Mean		Optimal Level Threshold		Allowable Mean	
$\lambda_{\sigma_{c}{}_{1}}^{*}$	0.3558	${\tilde{\sigma }}_{c_{10}}$	677.92	$\lambda_{\sigma_{c}{}_{2}}^{*}$	0.6008	${\tilde{\sigma }}_{c_{20}}$	654.94
$\lambda_{\varepsilon c_{1}}^{*}$	0.8051	${\tilde{\varepsilon }}_{c_{10}}$	2.010824	$\lambda_{\varepsilon c_{2}}^{*}$	0.8516	${\tilde{\varepsilon }}_{c_{20}}$	1.0076991
$\lambda_{Dc_{1}}^{*}$	0.6720	${\tilde{D}}_{c_{10}}$	0.2039	$\lambda_{Dc_{2}}^{*}$	0.1608	${\tilde{D}}_{c_{20}}$	0.2041
$\lambda_{Df_{1}}^{*}$	0.0260	${\tilde{D}}_{f_{10}}$	0.96363	$\lambda_{Df_{2}}^{*}$	0.8392	${\tilde{D}}_{f_{20}}$	0.90895

**Table 5:** Optimized results of a bladed disk.

Design Variable	Original Data	Optimization Results
*ω*, rad·s^−1^	1168	1055.1
*T,* K	873.15	788.15

**Table 6:** Dynamic probabilistic computational results with different methods. FMERSM: fuzzy multi-extremum response surface method, MC: Monte Carlo.

Number of Samples	Computational Time, s		Reliability Degree %		Precision of FMERSM
	MC Method	FMERSM	MC Method	FMERSM	
10^2^	32400	0.203	99	98.6	0.996
10^3^	72000	0.279	99.7	99.5	0.998
10^4^	432000	0.437	99.83	99.60	0.9977
10^5^	-	4.43	-	99.962	-

**Table 7:** Results of bladed disk optimization design with different methods.

Objective Function	Before Optimization	MCM		FMERSM	
		After Optimization	Reduction	After Optimization	Reduction
$\sigma_{c_{1}}$, MPa	607.92	548.66	9.8%	487.08	19.9%
$\sigma_{c_{2}}$, MPa	454.94	423.03	7%	368.8	18.93%
$\varepsilon_{c_{1}}$, m/m	0.010824	0.001298	88%	0.0073988	31.64%
$\varepsilon_{c_{2}}$, m/m	0.0076991	0.0076629	0.47%	0.0065652	14.77%
*Df_1_*	0.36363	0.30452	16.25%	0.24822	31.74%
*Df_2_*	0.40859	0.39375	4.52%	0.29315	28.3%
$D_{c_{1}}$	0.0039	0.0036	7.69%	0.0025	35.9%
$D_{c_{2}}$	0.0041	0.00409	0.24%	0.0032	21.95%
*R*	95	99.515	-	99.635	-
